# Enhanced Efficiency of the Microsomal Prostaglandin E_2_ Synthase‑1 Inhibitor AGU661 in Human Whole Blood by Encapsulation into PLGA-Based Nanoparticles

**DOI:** 10.1021/acs.molpharmaceut.5c00766

**Published:** 2025-10-03

**Authors:** Philipp Dahlke, Paul M. Jordan, Lea C. Klepsch, Azize Gizem Ergül, Steffi Stumpf, Stephanie Hoeppener, Antje Vollrath, Burcu Çalışkan, Erden Banoglu, Ulrich S. Schubert, Oliver Werz

**Affiliations:** † Department of Pharmaceutical/Medicinal Chemistry, Institute of Pharmacy, 9378 Friedrich Schiller University Jena, Philosophenweg 14, 07743 Jena, Germany; ‡ Jena Center for Soft Matter (JCSM), 9378 Friedrich Schiller University Jena, Philosophenweg 7, 07743 Jena, Germany; § Laboratory of Organic and Macromolecular Chemistry (IOMC), 9378 Friedrich Schiller University Jena, Humboldtstraße 10, 07743 Jena, Germany; ⊥ Department of Pharmaceutical Chemistry, Faculty of Pharmacy, 37511 Gazi University, Yenimahalle 06560 Ankara Turkey

**Keywords:** Inflammation, lipid mediators, oxylipins, prostaglandin, mPGES-1, nanoparticles

## Abstract

Microsomal prostaglandin E_2_ synthase 1 (mPGES-1) is a promising target for treating chronic inflammatory diseases and pain. The novel benzimidazole derivative AGU661 exhibits strong potency as a mPGES-1 inhibitor in a cell-free assay of prostaglandin E_2_ (PGE_2_) formation with IC_50_ = 0.22 nM. Comprehensive lipid mediator (LM) metabololipidomics with activated human monocytes and M1- and M2-monocyte-derived macrophages revealed high potency of AGU661 in intact cells with excellent selectivity for suppressing PGE_2_ among the broad spectrum of LMs. AGU661 possesses unfavorable physicochemical properties with poor metabolic stability and strong plasma protein binding tendencies, and thus, the compound lost efficiency in complex biological systems like blood. These hurdles could be overcome and the efficiency could be improved by encapsulation of AGU661 into poly­(lactic-*co*-glycolic acid) (PLGA)-based nanoparticles using nanoprecipitation. In comparison to free drug (IC_50_ > 100 nM), encapsulated AGU661 in PLGA-based solid nanoparticles revealed significantly enhanced potency in human whole blood (IC_50_ = 1.5 nM). Conclusively, we demonstrate that encapsulation of the mPGES-1 inhibitor AGU661 into polymer-based PLGA nanoparticles represents a suitable approach to improve the anti-inflammatory potential by enhancing its efficiency in complex biological settings like blood.

## Graphical abstract

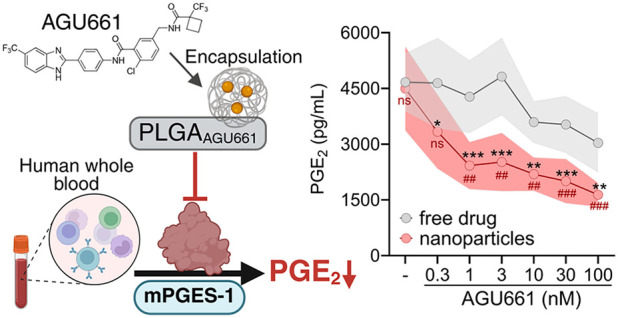

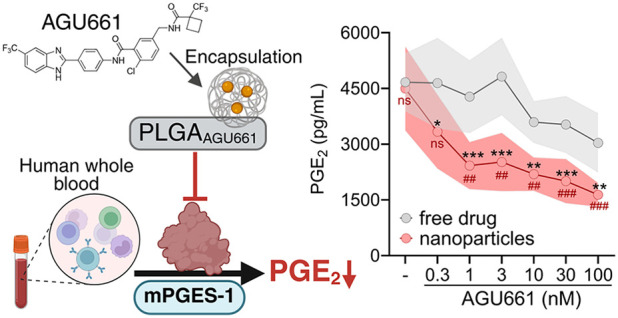

## 1 Introduction

Inflammation is the host’s physiological reaction in response to damage in order to combat harmful invaders and to repair damaged tissue. However, if inflammation is deregulated, persists, and the body cannot return to homeostasis, chronic inflammatory diseases such as arthritis, Alzheimer’s disease, or atherosclerosis may develop.^1^ Inflammation is initiated and maintained by pro-inflammatory lipid mediators (LM), such as prostaglandins (PG) and leukotrienes (LT) that are biosynthesized from arachidonic acid (AA). For PG formation, AA is first converted by cyclooxygenase-1 and -2 (COX-1/2) to the intermediate prostaglandin H_2_ (PGH_2_), which is subsequently metabolized by specific PG synthases into different bioactive prostanoids.^2^ PGE_2_ that is substantially produced by microsomal prostaglandin E_2_ synthase-1 (mPGES-1), is a key player in inflammation, mediating pain and fever, and increases vascular permeability.^3^ In the last two decades, mPGES-1 has become a well-studied target and mPGES-1 inhibitors are considered as promising candidates for a new class of anti-inflammatory drugs.^4,5^ Despite several candidates being tested in preclinical settings and phase 2 trials, no mPGES-1 inhibitor has yet reached the market.^6–8^ The reasons for this stagnant development are low water solubility and poor bioavailability (i.e., strong plasma protein binding) of drug candidates and also difficulties in preclinical testing because of interspecies differences in the amino acid sequence of mPGES-1.^9^ The motivation behind the extensive search for new mPGES-1 inhibitors is to supersede traditional nonsteroidal anti-inflammatory drugs (NSAIDs). These NSAIDs that are COX inhibitors, are still the predominant therapeutics for treating inflammation, although they exert several adverse on-target side effects and cause delayed inflammation resolution.^10^

Recently, we presented novel benzimidazole derivatives as potent mPGES-1 inhibitors with IC_50_ values in the 1-digit nanomolar range.^11^ Thus, AGU654 (2-Chloro-N-(4-(5-(tri­fluoro­methyl)-1H-benz­imidazol-2-yl)­phenyl)-5-((1-(trifluoro­methyl)­cyclo­propane-1-carbox­amido)­methyl)­benzamide) with an IC_50_ for mPGES-1 of 2.9 nM demonstrated potent anti-inflammatory effectiveness in a carrageenan-induced edema model in guinea pigs and reduced fever following LPS induction. AGU661 from this series carries an 1-(trifluoromethyl)-cyclobutyl residue with greater potency (IC_50_ = 0.22 nM) against isolated mPGES-1, but it exhibited poor metabolic stability in liver microsomes and achieved only very low plasma levels in guinea pigs.^11^ Nevertheless, in order to exploit the high potency of AGU661 against mPGES-1, we attempted to encapsulate the drug into solid nanoparticles (NPs) as drug delivery system to overcome the physicochemical/pharmacokinetic disadvantages and thus to improve its efficiency under pathophysiological relevant conditions.^12^ Various types of NPs have been developed as drug delivery systems, including those based on lipids, polymers, liposomes, and peptides.^13,14^

For hydrophobic drugs such as AGU661, polymeric NPs provide high encapsulation efficiency and drug-loading capacity, while enhancing overall stability by embedding and protecting the drug within the polymer matrix.^15^ Among the polymers extensively investigated for drug delivery, poly­(lactic-*co*-glycolic acid) (PLGA) is particularly noteworthy and was also employed in this study. PLGA is a well-studied, biocompatible polymer that degrades into glycolic and lactic acid and is approved by the U.S. Food and Drug Administration (FDA) and the European Medicines Agency (EMA) for medical applications, making it the most popular and efficient polymer for drug delivery.^16^ Importantly, PLGA can be synthesized with different molecular weights, lactic acid-to-glycolic acid ratios, and terminal functional groups (e.g., carboxyl or ester), which, in combination with formulation parameters, strongly influence NP stability, degradation kinetics, and drug release profiles.^17^

Here, we comprehensively characterized AGU661 as a potent mPGES-1 inhibitor in cell-based models, showing that it lowers PGE_2_ formation in human pro-inflammatory M1 macrophages and activated monocytes without affecting other LM pathways. Intriguingly, encapsulated AGU661 was far more efficient in suppressing PGE_2_ formation in an LPS-primed human whole blood model than the free drug.

## 2 Materials and Methods

### 2.1 Materials

Purified water was obtained from a GenPure ultrapure water purification system (Thermo Scientific) and was used at all stages of the NP preparation, purification, and characterization studies. The Resomer RG 502 H (PLGA, Mw 7,000 to 17,000 g mol^–1^, lactide:glycolide 50:50, acid terminated), poly­(vinyl alcohol) (PVA, Mowiol 4–88, degree of hydrolysis of 86.7–88.7 mol %, Mw 31,000 g mol^–1^) and dimethyl sulfoxide (DMSO, anhydrous ≥ 99.9%) were obtained from Sigma-Aldrich (Munich, Germany). Acetone (99+%, extra pure) was purchased from Acros Organics and was received from Carl Roth GmbH. AGU661 was synthesized based on the established procedure.^11^ Indomethacin and all other fine chemicals were obtained from Sigma-Aldrich unless stated otherwise.

### 2.2 Isolation of Polymorphonuclear Leukocytes and Monocytes and Generation of Monocyte-Derived Macrophages

Leukocyte concentrates derived from freshly withdrawn blood (16 I.E. heparin/mL blood) of healthy adult male and female volunteers (18 to 65 years, without details about ancestry, race, or ethnicity) were provided by the Department of Transfusion Medicine at the University Hospital of Jena, Germany. The experimental procedures were approved by the local ethical committee (approval no. 5050-01/17) and were performed in accordance with the guidelines and regulations. Voluntary donors agreed to use the software via written consent. Following an established protocol,^18^ polymorphonuclear leukocytes (PMNL) and peripheral blood mononuclear cells (PBMC) were isolated using density gradient centrifugation after addition of a lymphocyte separation medium (Histopaque-1077, Sigma-Aldrich) after sedimentation of erythrocytes by dextran. PMNL were purified by hypotonic lysis of erythrocytes with pure water and afterward washed twice with PBS (pH 7.4) and finally resuspended in PBS (pH 7.4). To isolate monocytes, PBMC were seeded in cell culture flasks (Greiner Bio-one, Frickenhausen, Germany) in PBS pH 7.4 with CaCl_2_ and MgCl_2_ (Sigma-Aldrich). After 1 h at 37 °C and 5% CO_2_ for adherence of the monocytes, the medium was discarded and replaced with RPMI 1640 (Sigma-Aldrich) containing 10% (v/v) heat-inactivated fetal calf serum (FCS), 2 mmol/L glutamine (Biochrom/Merck, Berlin, Germany), 100 U/mL penicillin, and100 μg/mL streptomycin (Biochrom/Merck). For differentiation toward macrophages, the monocytes were kept in RPMI 1640 supplemented with 10% (v/v) FCS, 2 mmol/L glutamine, 100 U/mL penicillin, and 100 μg/mL streptomycin for 6 days with either 20 ng/mL GM-CSF (Peprotech, Hamburg, Germany) for M0_GM‑CSF_ or 20 ng/mL M-CSF (Peprotech) for M0_M‑CSF_ differentiation. Afterward, M0_GM‑CSF_ were incubated with 100 ng/mL LPS and 20 ng/mL IFN-γ (Peprotech) for another 24 h to obtain M1-monocyte-derived macrophages (MDM) and M0_M‑CSF_ were incubated with 20 ng/mL IL-4 (Peprotech) for 48 h to generate M2-MDM.

### 2.3 Incubation of Monocytes and MDM

Monocytes (5 × 10^6^) were seeded in PBS pH 7.4 with MgCl_2_ and CaCl_2_ for 1.5 h at 37 °C and 5% (v/v) CO_2_ after isolation in 12-well plates and washed three times with PBS pH 7.4. Subsequently, cells were kept in RPMI 1640 (Sigma-Aldrich) containing 5% (v/v) heat-inactivated FCS, 2 mmol/L glutamine (Biochrom/Merck), 100 U/mL penicillin, and 100 μg/mL streptomycin (Biochrom/Merck). After preincubation for 15 min with vehicle (DMSO 0.1%), indicated concentrations of AGU661 or indomethacin (10 μM) as positive control, monocytes were stimulated with 100 ng/mL LPS for 24 h. For MDM incubations, after polarization to M1- or M2-MDM, medium was discarded, and PBS plus 1 mM CaCl_2_ was given to the cells. After preincubation for 15 min with vehicle (0.1% DMSO), indicated concentrations of AGU661, or indomethacin (10 μM) as positive control, cells were stimulated with 1% *Staphylococcus aureus*-conditioned medium (SACM) for 90 min at 37 °C and 5% CO_2_. SACM was generated as previously described.^18^ Incubation was stopped by adding methanol, and LMs were analyzed as described by LM metabololipidomics below.

### 2.4 Incubation of PMNL and RP-HPLC Analysis of 5-Lipoxygenase Products

PMNL (5 × 10^6^) were preincubated with vehicle (0.1% DMSO), AGU661, or zileuton (Cayman Chemical) in PBS plus 1 mM CaCl_2_ and 0.1% glucose at 37 °C in a water bath for 15 min and stimulated with 2.5 μM A23187 (Cayman Chemical/BiomolGmbH, Hamburg, Germany) for 10 min, and the incubation was stopped with 1 mL of ice-cold methanol containing 200 ng/mL PGB_1_ as internal standard. Samples were subjected to SPE, and the formed 5-lipoxygenase (LOX) products (i.e., leukotriene B_4_ (LTB_4_), trans-isomers of LTB_4_, 5-hydroxyeicosatetraenoic acid (5-HETE)) were separated and analyzed by reverse-phase high-performance liquid chromatography (RP-HPLC) as previously described.^19^

### 2.5 Isolation and Treatment of Murine Peritoneal Macrophages

After euthanizing mice with CO_2_ inhalation in accordance with standard operation procedures at the Leibnitz Institute on Aging – FLI or the Bioinstrumentezentrum, 10 mL of ice-cold RPMI 1640 (Sigma-Aldrich) was injected into the peritoneal cavity and the peritoneal lavage was recovered, according to a recently published protocol.^20^ Centrifugation at 4 °C for 10 min at 2000*g* was carried out, supernatant was wasted, and cells were resuspended in 1 mL of RPMI 1640 (Sigma-Aldrich) containing 5% (v/v) heat-inactivated FCS, 2 mmol/L glutamine (Biochrom/Merck), 100 U/mL penicillin, and100 μg/mL streptomycin (Biochrom/Merck). Cells (0.5 × 10^6^) were seeded in 12-well plates and treated with 100 ng/mL LPS for 24 h. After 15 min of preincubation, cells were stimulated for 90 min with 1% SACM in PBS plus 1 mM CaCl_2_. Incubation was stopped by adding methanol, and LMs were analyzed as described in the LM metabololipomics section below.

### 2.6 Analysis of LM Formation in Stimulated Human Whole Blood

AGU661 and AGU661-loaded NPs were tested in freshly withdrawn peripheral Li-heparin blood from human healthy donors who had not received any anti-inflammatory treatment for the last 10 days, provided by the Institute of Transfusion Medicine, Jena University Hospital. Experiments were performed following an established protocol.^21^ Preincubation of 0.5 mL of whole blood with vehicle (0.1% DMSO or blank-PLGA-NPs), AGU661, or AGU661-loaded NPs for 15 min was followed by 24 h stimulation with 1 μg/mL LPS at 37 °C and 5% CO_2_. Afterward, 3% SACM was added for 90 min of stimulation. Extraction of LMs was conducted with SPE after centrifugation at 4 °C with 3000 × g.

### 2.7 LM Metabololipidomics

After the incubation, the supernatants were transferred to 2 mL of ice-cold methanol containing 10 μL of deuterium-labeled internal standards (200 nM d8–5S-HETE, *d*_4_-LTB_4_, d5-LXA_4_, d5-RvD2, d4-PGE_2_, and 10 μM d8-AA; Cayman Chemical/BiomolGmbH) to facilitate quantification and sample recovery. Sample purification was accomplished as published recently.^18^ In brief, samples were kept at −20 °C for at least 60 min to allow protein precipitation. Centrifugation (1200*g*, 4 °C, 10 min) and acidification using 9 mL acidified water (final pH 3.5) was followed by solid phase extraction (SPE). Solid phase cartridges (Sep-PakVac 6 cm^3^ 500 mg/6 mL C18; Waters, Milford, MA) were washed with 6 mL of methanol and 2 mL of H_2_O before samples were loaded onto columns. After washing with 6 mL of H_2_O and additional 6 mL of *n*-hexane, LM were eluted with 6 mL of methyl formate. Finally, the samples were evaporated using an evaporation system (TurboVap LV, Biotage, Uppsala, Sweden) and resuspended in 100 μL of a methanol–water mixture (50/50, v/v) for UPLC-MS/MS automated injections. For UPLC-MS/MS an Acquity UPLC system (Waters, Eschborn, Germany) and a QTrap 5500 Mass Spectrometer (Sciex, Framingham, MA, USA) equipped with an electrospray ionization source exactly was employed, as described before.^18^

### 2.8 Determination of Extracellular Cytokine Levels

Monocytes (1 × 10^6^) were seeded in RPMI 1640 (Thermo Fisher Scientific, Schwerte, Germany) containing 5% (v/v) heat-inactivated FCS, 2 mmol/L glutamine (Biochrom/Merck), 100 U/mL penicillin, and 100 μg/mL streptomycin (Biochrom/Merck) and treated with vehicle (0.1% DMSO) or AGU661 at the indicated concentrations 30 min prior to stimulation with 1 μg/mL LPS for 6 h at 37 °C and 5% CO_2_. Extracellular levels of TNFα, IL-6, and IL-1β were measured in cell-free supernatants using enzyme-linked immunosorbent assay (ELISA), according to manufacturer’s instructions (R&D Systems, Minneapolis, Minnesota).

### 2.9 Expression, Purification and Activity Assay of Human Recombinant 5-LOX

As described before, *Escherichia coli* (*E. coli*) was transformed with pT3–5-LO plasmid at 30 °C overnight for expression of human recombinant 5-LOX.^22^ The bacteria were treated with lysis buffer containing triethanolamine (50 mM, pH 8.0, Cayman Chemical/BiomolGmbH), EDTA (5 mM, Sigma-Aldrich), phenylmethanesulfonyl fluoride (1 mM, Cayman Chemical/BiomolGmbH), soybean trypsin inhibitor (60 μg/mL, Sigma-Aldrich), dithiothreitol (2 mM), and lysozyme (1 mg/mL) before sonification (3 × 15 s) to obtain cell lysates. 5-LOX was subsequently purified by ATP affinity chromatography as described.^22^ Then, 0.5 μg of 5-LOX was preincubated for 15 min with vehicle (0.1% DMSO), AGU661 or zileuton (Cayman Chemical) in PBS plus 1 mM EDTA and stimulated with 20 μM AA and 2 mM CaCl_2_ for 10 min at 37 °C. The reaction was stopped by addition of 1 mL ice-cold methanol containing 200 ng PGB_1_ as internal standard, and 5-LOX products (trans-isomers of LTB_4_ and 5-HETE) were analyzed by RP-HPLC as described previously.^23^

### 2.10 Activity Assays of Isolated COX-1 and COX-2

Purified ovine COX-1 (50 units, Cayman Chemical/Biomol GmbH) or human recombinant COX-2 (20 units, Cayman Chemical/Biomol GmbH) were preincubated with the test compounds in reaction buffer (1 mL; 100 mM Tris buffer pH 8, 5 mM glutathione, 5 μM hemoglobin, and 100 μM EDTA) for 5 min at 4 °C, followed by 1 min at 37 °C. Then, AA (COX-1: 5 μM; COX-1: 2 μM) was added as the substrate for the COX reaction. COX-derived 12-hydroxyheptadecatrienoic acid (12-HHT) was extracted after 5 min and analyzed by RP-HPLC as described.^24^

### 2.11 Cell Viability Assays

Cytotoxicity was assessed by a lactate dehydrogenase (LDH) assay and by a 3-(4,5-dimethyl­thiazol-2-yl)-2,5-diphenyl­tetrazolium bromide (MTT) assay using two different culture media: PBS containing 1 mM CaCl_2_ for 3 h for LDH assay, and RPMI 1640 supplemented with 10% (v/v) heat-inactivated fetal calf serum, 2 mM glutamine, 100 U/mL penicillin, and 100 μg/mL streptomycin for the MTT assay. Cell membrane integrity was assessed via measuring of LDH release (CytoTox 96 Non-Radioactive Cytotoxicity assay, Promega, Mannheim, Germany) following the manufacturer’s instructions. After 3 h of incubation of M0_GM‑CSF_ with 0.1% DMSO as vehicle, AGU661 at 1, 10, 100, or 1000 nM, or triton X-100 (0.9% v/v) as positive control, cells were centrifuged at 400*g* for 5 min at 4 °C, and supernatants were transferred to a 96 well-plate. Absorbance was measured at 490 nm by using a NOVOstar microplate reader (BMG Labtechnologies GmbH, Offenburg, Germany). M0_GM‑CSF_ viability after 48 h of incubation with 0.1% DMSO as vehicle, AGU661 or staurosporine (abcam; positive control) was assessed using MTT assay (MTT, 5 mg/mL, 20 μL; Sigma-Aldrich). The formazan product was solubilized with sodium dodecyl sulfate (SDS, 10% in 20 mM HCl), and the absorbance was monitored at 570 nm (Multiskan Spectrum microplate reader, Thermo Fisher Scientific).

### 2.12 Formulation of Blank NPs (PLGA) and AGU661-Loaded NPs (PLGA_AGU661_)

PLGA (15 mg) was dissolved in 1 mL of acetone, and AGU661 was dissolved in DMSO (10 mg/mL). For the drug-loaded NPs, the polymer solution (1 mL) was mixed with 2.3 μL of the AGU661 stock solution. A glass vial was prepared with 8 mL of 0.3% (w/v) aqueous PVA. Then, the polymer solutions were transferred to a 2 mL syringe with a 21G × 43/4 (0.8 × 120 mm) cannula, which was mounted on a syringe pump (Aladdin AL1000–220, World Precision Instruments, Friedberg, Germany). The cannula was bent at a 90° angle and placed in the glass vial, touching the glass wall. The polymer solution was injected into the aqueous phase at a flow rate of 2 mL/min, while the solution was stirred at 800 rpm. The resulting solution was continuously stirred at 800 rpm under a fume hood overnight to evaporate the acetone. Subsequently, the NPs were purified by centrifugation using a 5804 R centrifuge (Eppendorf, Wesseling-Berzdorf, Germany) at 11,000 rpm for 1 h at 20 °C. The resulting pellet was resuspended in 2 mL of Milli-Q water via pipetting up and down 5 to 10 times, and the dispersions were stored overnight at 4 °C. The final NP concentrations were determined by freeze-drying defined volumes of 0.25 mL with a Lyophilizer Christ Alpha 2–4 LD plus (Germany), and the sample mass was assessed using an XPR10 microbalance (Mettler Toledo, Switzerland). Detailed formulation parameters for PLGA and PLGA_AGU661_ are shown in Table 1.

**Table 1:** Particle Characteristics^a^

		**After purification**			**After reconstitution w/o PVA**		**After reconstitution w/PVA**	
**Formulation**	**LC [%]**	**d**_**h**_ **[nm]**	**PDI**	**Zeta potential MQ [mV]**	**d**_**h**_ **[nm]**	**PDI**	**d**_**h**_ **[nm]**	**PDI**
PLGA_AGU661_	0.11 ± 0.06	181 ± 13	0.04 ± 0.02	–31.3 ± 7.5	211 ± 13	0.08 ± 0.04	195 ± 10	0.06 ± 0.04
PLGA	-	180 ± 6	0.04 ± 0.02	–31.3 ± 6.6	218 ± 6	0.10 ± 0.05	226 ± 30	0.17 ± 0.10

a Loading capacity (LC) in % was assessed by UV–vis measurements of lyophilized particles. Z-Average hydrodynamic diameter (d_h_) and polydispersity index (PDI) obtained by DLS in water. Zeta potential was measured postcentrifugation in water. All values are shown as mean ± SD with single values with PLGA_AGU661_ *n* = 4, and PLGA *n* = 3.

### 2.13 Dynamic and Electrophoretic Light Scattering (DLS & ELS)

PLGA and PLGA_AGU661_ NP dispersions were diluted to a ratio of 1:100 in pure water and analyzed regarding their size distributions and zeta potentials within polystyrene microcuvettes (Brand GmbH + Co KG, Wertheim, Germany) at a temperature of 25 °C using the Zetasizer Ultra (Malvern Panalytical Ltd., Malvern, United Kingdom). The instrument operates with a laser wavelength of 633 nm and measures the particles at a backscattering angle of 174.7°. The samples were measured five times with each assessment consisting of 15 runs lasting 1.68 s. The hydrodynamic diameter (*d*_H_) and polydispersity index (PDI) of the particles are calculated using the intensity-weighted size distribution. The *d*_H_ and PDI were controlled at three different stages within the formulation: (i) after the evaporation of the organic solvent, (ii) after centrifugation and subsequent resuspension in water, and (iii) postlyophilization and resuspension in water. The zeta potential was determined after centrifugation and resuspension in water also with a dilution of 1:100 in pure water using a DTS1070 capillary cuvette and with three automated measurements per samples.

### 2.14 UV–vis Spectroscopy

To determine the drug load, UV–vis measurements were performed by using the Infinite M200 Pro plate reader (Tecan Group, Männedorf, Switzerland). The lyophilized particle samples were dissolved in DMSO and stirred for 15 min. Following this, 100 μL of both undiluted and diluted solutions were measured using a Hellma quartz 96-well plate. The AGU661 absorbance was measured at λ_ex_ = 325 nm, with 3 × 3 multiple readings per well and a well border of 2000 μm. The loading capacity (LC) was calculated by dividing the recovered drug mass by the recovered particle mass and then multiplying the result by 100. The encapsulation efficiency (EE) was calculated by dividing the determined LC by the initial used wt % of drug added to the formulation, multiplied by 100.^21^

### 2.15 Scanning Electron Microscopy

Following established protocols, imaging of the NPs was performed using a Sigma VP field emission scanning electron microscope (Carl-Zeiss AG, Germany). Micrographs were acquired using the InLens detector at an acceleration voltage of 6 kV. Diluted NP samples were pipetted onto mica substrates and allowed to air-dry. Prior to imaging, the samples were sputter-coated with a 4 nm layer of platinum using the CCU-010 HV sputter coater (Safematic GmbH, Switzerland).^25^

### 2.16 Poly­(vinyl alcohol) (PVA)-Assay

The residual PVA content (% w/w) in the freeze-dried NPs was quantified using UV–vis spectroscopy based on the formation of a PVA-iodine complex. A 0.25 mL freeze-dried aliquot was resuspended in 1 mL of pure water. Then, 90 μL of the suspension was transferred into a VWR Tissue Culture 96-well-F plate (VWR, Darmstadt, Germany), with each sample prepared in triplicate. Next, 20 μL of 1 M sodium hydroxide was added to each well, and the mixture was incubated at room temperature for 15 min at 850 rpm in a Grant Bio PCMT Thermoshaker was used to hydrolyze the NP matrix. After incubation, 20 μL of 1 M hydrochloric acid was added to neutralize the solution. Complexation was initiated by adding 60 μL of 0.65 M boric acid and 10 μL of Lugol’s solution. Finally, sample absorption was measured at λ = 650 nm within 15 min using a plate reader.^26^

### 2.17 Statistics

Results are expressed as mean ± standard error of the mean (SEM) or standard deviation (SD), as indicated, of *n* observations, where n represents the number of experiments with cells from separate donors, performed on different days. Data sets were analyzed by GraphPad Prism 10.1.0 (GraphPad, La Jolla, CA, USA). One-way ANOVA and Tukey’s multiple comparisons test were used for statistical analysis in case that one different independent variable influences one continuous dependent variable. Two-way ANOVA was used for statistical analysis in case of two different categorical independent variables influence one continuous dependent variable, as indicated. The criterion for statistical significance is *p < 0.05; **p < 0.01; ***p < 0.001.

## 3 Results

### 3.1 AGU661 Specifically Suppresses Formation of PGE_2_ in Pro-inflammatory Macrophages

Previous results showed that AGU661 effectively inhibits the enzyme activity of mPGES-1 in microsomes in a cell-free assay (IC_50_ = 0.22 nM),^11^ but whether AGU661 also interferes with PGE_2_ formation in intact cells is unknown. We employed human M1- and M2-MDM, which are well-suitable test systems for LM biosynthesis inhibitor studies, to investigate this aspect, considering the selectivity over other branches in the complex LM network.^27^ To elicit formation of a broad spectrum of pro-inflammatory LM, we used M1-MDM that respond to *S. aureus-*conditioned medium (SACM that contains exotoxins) with marked PG and LT formation, whereas M2-MDM generate substantial 15-LOX-derived LM including specialized pro-resolving mediators (SPM).^28^ AGU661 (100 nM) differentially modulated LM signature profiles in SACM-stimulated M1- and M2-MDM within 90 min, as displayed in radar plots (Figure 1a). In detail, in M1-MDM that express high levels of COX-2 and mPGES-1,^29^ AGU661 reduced the massive PGE_2_ formation in a concentration-dependent manner, starting with significant inhibition at 10 nM and almost complete suppression at 100 and 1000 nM, while other prostanoids, such as PGD_2_ and TXB_2_, as well as the 5-LOX-derived LTB_4_ were not impaired (Figure 1a, b). In contrast, in M2-MDM that do not express mPGES-1,^29^ AGU661 failed to significantly suppress PGE_2_ (as expected) or other LM branches such as 5- or 15-LOX-mediated LT and SPM formation (Figure 1a, c). The NSAID indomethacin, an unselective COX-1/2 inhibitor, was used as a control in these experiments, suppressing all COX-mediated products (i.e., PGE_2_, PGD_2_ and TXB_2_), as expected (Figure 1b, c).

**Figure 1:**
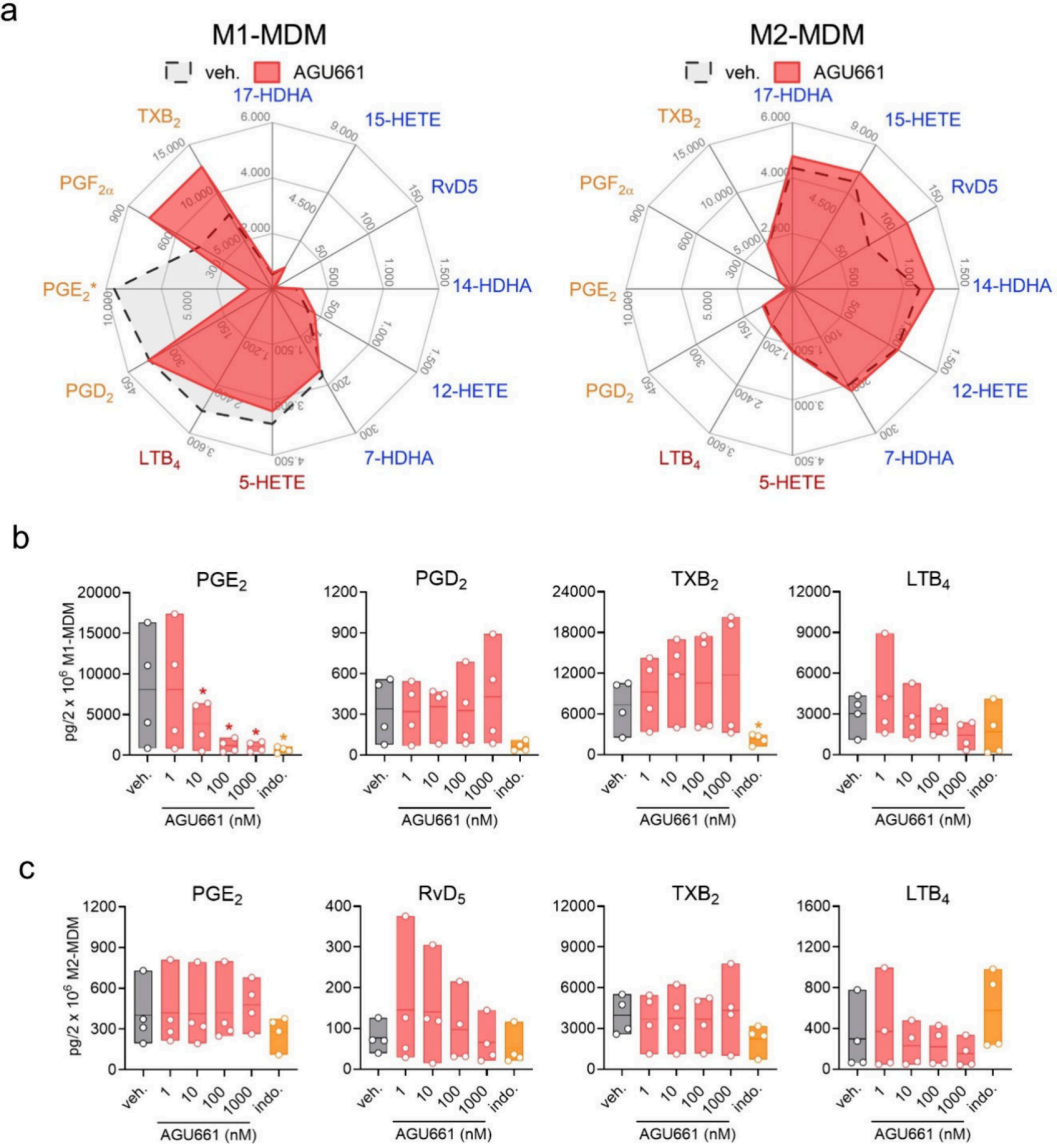
AGU661 modulates exotoxin-induced LM formation in human macrophages. M1- and M2-MDM (2 × 10^6^) were resuspended in 1 mL PBS containing 1 mM CaCl_2_, preincubated with vehicle (veh., 0.1% DMSO), the indicated concentrations of AGU661 or 10 μM indomethacin (indo.) as reference drug for 15 min, and stimulated with 1% SACM for 90 min at 37 °C and 5% CO_2_. Supernatants were collected, and formed LM were analyzed by UPLC-MS/MS. (a) Results are presented as pg/2 × 10^6^ M1- or M2-MDM in a radar chart, indicating the impact of 100 nM AGU661. (b,c) Results are presented as pg/2 × 10^6^ cells given as mean with single values of (b) M1-MDM and (c) M2-MDM, *n* = 4. For statistical analysis, data were log-transformed, and one-way ANOVA with Dunnett’s multiple comparison test against veh. was performed; * *p* < 0.05.

AGU661 did not affect cell viability in MDM, as displayed in short-term experiments (3 h), assessed by LDH release assay, and in long-term experiments (48 h), evaluated by MTT assay (Figure S1). Interspecies differences in the primary amino acid sequence of mPGES-1 hamper the development of inhibitors since most candidates that inhibit human mPEGS-1 do not bind mouse or rat mPGES-1.^9^ We thus examined if AGU661 follows the same pattern by using murine peritoneal macrophages (PM) stimulated with LPS for 24 h and then treated with AGU661 and 10 μM indomethacin followed by SACM (1%) stimulation. We measured the formation of a broad spectrum of LMs, including PGE_2_ and other PGs, LTs, and SPMs, which were not significantly altered by AGU661, indicating that AGU661 does not inhibit the murine ortholog of mPGES-1 (Figure S2).

### 3.2 AGU661 Specifically Suppresses Formation of PGE_2_ in Circulating Innate Immune Cells

Next, we investigated whether AGU661 is also prominent in suppressing PGE_2_ formation in circulating innate immune cells such as monocytes, which are present in human peripheral blood.

Freshly isolated monocytes were preincubated with AGU661, and then stimulated with 100 ng/mL LPS for 24 h in order to induce mPGES-1 and COX-2. PGE_2_ levels were strikingly suppressed by 50% at 1 nM AGU661; at higher concentrations of 100 and 1000 nM, minor inhibitory effects on LTB_4_, PGD_2_, and PGF_2α_ formation were evident (Figure 2a, b). To assess effects of AGU661 on pro-inflammatory cytokine secretion in monocytes, cells were pretreated with AGU661 for 15 min and stimulated with 1 μg/mL LPS for 6 h. LPS increased IL-1β, IL-6, and TNFα levels as expected, but these cytokines were not significantly altered by AGU661, while minor suppressive effects on IL-1β and IL-6 and were observed at 1000 nM (Figure 2c). Along these lines, we investigated whether AGU661 could inhibit other prominent enzymes in the LM network. We found no inhibition of 5-LOX in cell-free and cell-based (PMNL) assays by AGU661 (Figure 2d) and also no effect on isolated COX-1 and COX-2 up to 1000 nM (Figure 2e); zileuton (5-LOX) and indomethacin (COX-1/2) worked as expected (not shown).

**Figure 2:**
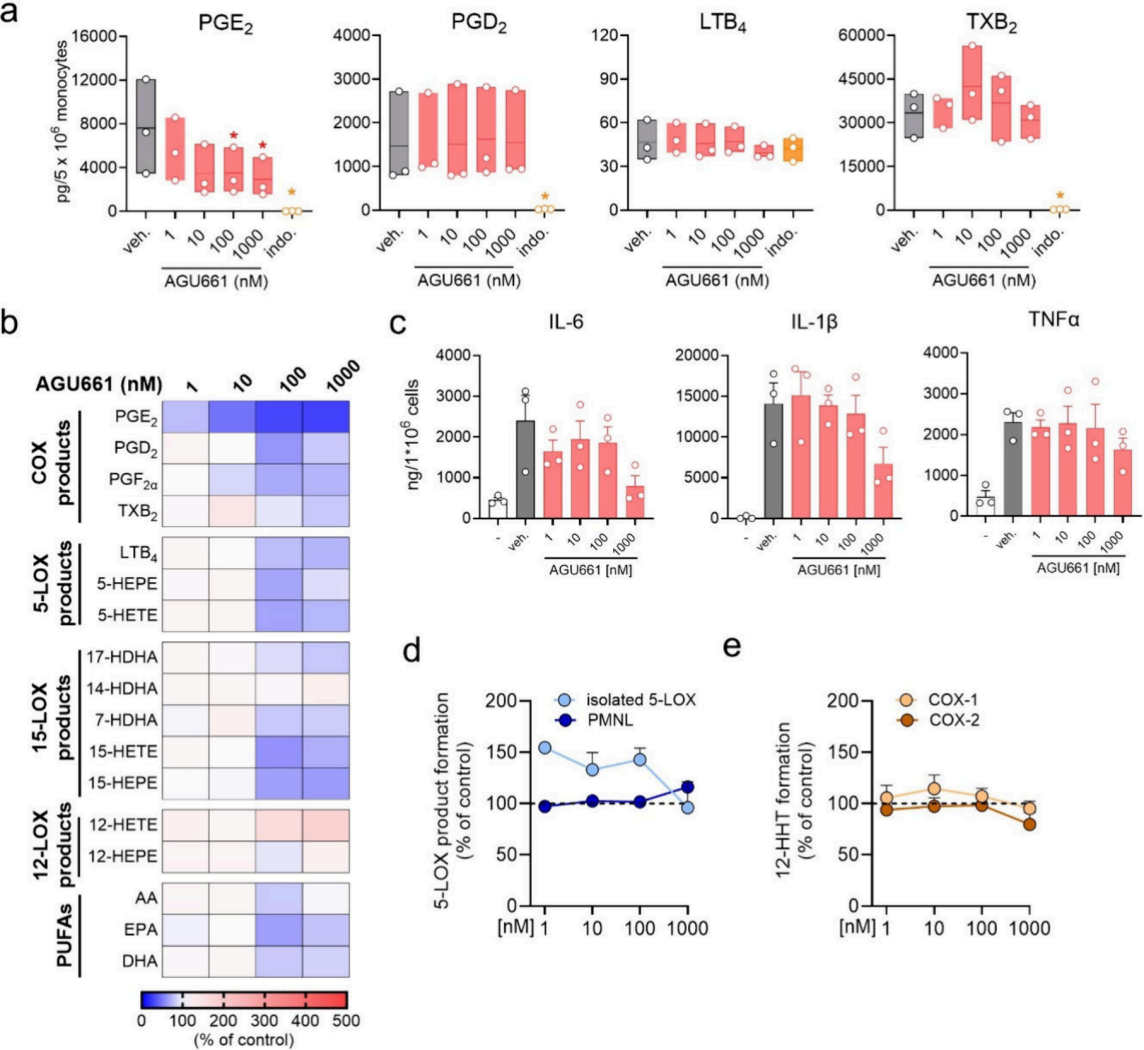
Modulation of LM biosynthesis and cytokine formation in monocytes by AGU661. (a, b) Human monocytes (5 × 10^6^) were preincubated with vehicle (veh., 0.1% DMSO), the indicated concentrations of AGU661 or with indomethacin (indo., 10 μM) for 15 min at 37 °C and 5% CO_2_ and then stimulated with 100 ng/mL LPS for 24 h. Supernatants were collected, and formed LM were analyzed by UPLC-MS/MS. (a) Results are presented as pg/5 × 10^6^ monocytes, given as mean with single values, *n* = 3. For statistical analysis data were log-transformed and one-way ANOVA with Dunnett’s multiple comparison test against veh. was performed; * *p* < 0.05. (b) Results are presented as % of control (stimulated cells = 100%) in a heatmap. (c) Monocytes (5 × 10^6^) were preincubated with vehicle (veh., 0.1% DMSO) or the indicated concentrations of AGU661 for 15 min at 37 °C and 5% CO_2_ and stimulated with 1 μg/mL LPS for 6 h. Supernatants were collected, and cytokines were measured by ELISA. Results are presented as ng/10^6^ monocytes + SEM with single values, *n* = 3. (d) Effect of AGU661 on 5-LOX activity. PMNL (5 × 10^6^) were resuspended in 1 mL PBS containing 1 mM CaCl_2_ and 0.1% glucose and preincubated with vehicle (veh., 0.1% DMSO) or the indicated concentrations of AGU661 for 15 min at 37 °C and stimulated with 2.5 μM A23187 for 10 min. Isolated human recombinant 5-LOX was incubated in PBS containing 1 mM EDTA and preincubated with vehicle (veh., 0.1% DMSO) or the indicated concentrations of AGU661 for 15 min at 4 °C. Incubations were prewarmed for 30 s and 2 mM CaCl_2_ and 20 μM AA were added and further incubated at 37 °C for 10 min. To determine 5-LOX activities, the incubations were stopped with ice-cold MeOH (containing PGB_1_), and leukotrienes and 5-HETE were extracted using SPE and analyzed with RP-HPLC. Results are shown as % of control + SEM, *n* = 3. (e) Isolated COX-1 and COX-2 were preincubated with vehicle (0.1% DMSO) or the indicated concentrations of AGU661 for 5 min at RT followed by 10 min at 37 °C with AA (for COX-1:5 μM AA; for COX-2:2 μM AA). The incubations were stopped with ice-cold MeOH (containing PGB_1_), and 12-HHT was extracted using SPE and analyzed with RP-HPLC. Results are shown as % of control + SEM *n* = 3.

### 3.3 Encapsulation of AGU661 into PLGA-Based Nanoparticles

One common drawback of many mPGES-1 inhibitors is their failure in more complex biosystems like in blood and tissues or *in vivo,* mainly due to marked plasma protein binding,^30^ and pilot studies indicated that this also applies to AGU661 that poorly inhibited PGE_2_ formation in human blood (see below, Figure 5). To improve the bioavailability and efficiency of AGU661 in this respect, we encapsulated the drug into solid polymeric NPs based on PLGA (LA:GA ratio 50:50, 7–17 kDa, and COOH terminated) using nanoprecipitation. The surfactant PVA (Mowiol 4–88, ∼31 kDa) was used as a stabilizer. The formulation parameter of PLGA and PLGA_AGU661_ NPs are shown in Table 1 and Table S1. All formulation characteristics of the blank NPs (PLGA) and AGU661-loaded NPs (PLGA_AGU661_) are shown in Table S2. The blank NPs (PLGA) and AGU661-loaded NPs (PLGA_AGU661_) resulted in uniformly distributed spherical-shaped NPs for both formulations, as observed by scanning electron microscopy (Figure 3a). The sizes for both NP systems were about 210 nm (Figure 3b) and PDI values of the formulations were below 0.1 (Figure 3c), indicating a unimodal particle dispersion (PDI < 0.2) (see intensity plots of the hydrodynamic diameter (Figure S3,S4).^31^ Intensity plots of the hydrodynamic diameters of the resuspended particles from DLS measurements are provided in Figure S5,S6. Both NP systems revealed negative surface charges (zeta potential) of below −30 mV in water (Figure 3d), suggesting a strong electrostatic repulsion of the particles against each other, indicating a stable formulation.^32^ Additionally, the zeta potential was measured in PBS and diluted sodium chloride solution, revealing a low negative surface charge (−1.8 mV for both; see Table S2). The NP loading capacity (LC) of 0.11% (encapsulation efficiency = 72.3%) was sufficient for performing favorable NP – cell ratio experiments due to the high potency of AGU661 (Figure 3e). Please note that higher amounts of AGU661 (e.g., up to 1%) were also encapsulated (Table S1, S2), but were not necessary for the biological assays. Furthermore, the reconstitution of the NPs after freeze-drying was investigated, showing that with and without the addition of the stabilizer PVA (10 μL of 0.3 wt % PVA aq solution), the dispersion remained stable with size values around 210 nm and PDI values below 0.1 after reconstitution (Table 1).

**Figure 3:**
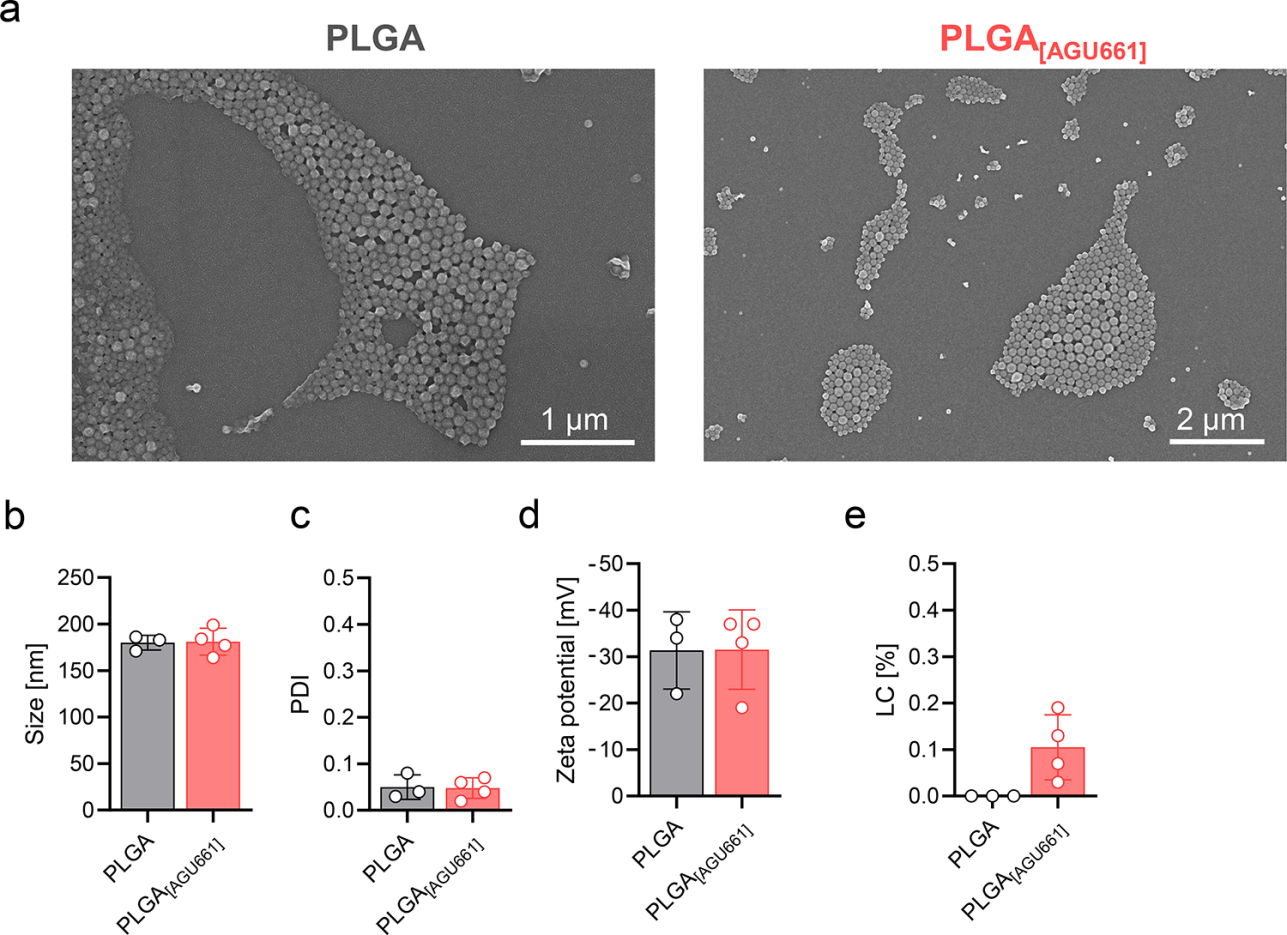
Physicochemical characterization of AGU661-loaded PGLA nanoparticles. (a) Images of the generated PLGA and PGLA_AGU661_ NPs obtained via SEM analysis. (b, c) Size (in nm) and size distribution of NPs were determined using DLS/ELS, results are shown as mean ± SD with single values. (d) Zeta potential was measured postcentrifugation and resuspension in water using a zetasizer, shown in mV as mean ± SD with single values. (e) Loading capacity (LC) in % was assessed by UV–vis measurements and shown as mean ± SD with single values.

### 3.4 Stability and Degradation of NPs

In order to develop a suitable nanocarrier that can protect the drug upon intravenous application from premature degradation, protein binding, and elimination from the body while simultaneously releasing the drug at the right time and at the right place, the nanocarrier should exhibit high stability but also reveal an optimal drug release profile. The selected PLGA polymer is widely used for drug-NP formulations.^33^ It degrades by hydrolysis of the ester bonds, breaking down into lactic and glycolic acid while undergoing different phases, e.g. the hydration phase, in which the polymer absorbs water, the initial and progressive degradation phase (along with drug release), and finally the polymer dissolution (complete drug release).^34^ It exhibits thereby very different degradation rates, depending on the lactide-to-glycolide ratio (LA:GA), molar mass and end group, but also on the applied formulation conditions and stabilizers.^17^ PLGA with a higher LA content is reported to be more stable, while the introduction of GA units reduces the crystallinity, which in turn increases the swelling behavior and water absorption leading to accelerated degradation. The selected PLGA inherited a 50:50 LA to GA ratio and is a fast-degrading PLGA polymer with a molar mass of 7 to 17,000 g mol^–1^. Additionally, it carries carboxyl end groups (COOH), which might further accelerate the hydrolysis of ester bonds in PLGA (autocatalytic effect).^34^ It is known that higher molar masses are more stable, which is attributed to the higher end-group density in low-molar-mass polymers, which enhances the contribution of end groups to degradation through autocatalytic processes. However, another important parameter for the stability or degradation of PLGA particles is the surrounding medium, its pH value, ionic strength, and temperature. Drug-loaded PLGA particles can remain stable in cold water (4 °C) for months, while in various media, including PBS or cell culture media at 37 °C, they can very quickly, i.e. within a few days, show instabilities such as aggregation or decay. Of course, the presence of enzymes such as esterases or proteinases can also degrade PLGA through enzymatic degradation.

To assess the stability of the NPs, PLGA_AGU661_ was incubated in PBS at 37 °C and measured *via* DLS to monitor changes in the count rate and hydrodynamic diameter over time using constant detector settings. As shown in Figure 4a, both the count rate and the particle size remained stable for up to 60 h, indicating colloidal stability under these conditions. Despite the relatively low surface charge of the particles in PBS, no aggregation was observed. To investigate NP degradation, proteinase K was chosen due to its broad substrate specificity. PLGA_AGU661_ was incubated with proteinase K at a fixed NP-to-enzyme mass ratio of 1:0.05 (Figure 4b). A progressive decrease in the count rate was observed, reflecting enzymatic degradation of the NPs. Notably, no increase in the hydrodynamic diameter was detected, suggesting that NP aggregation did not occur during the degradation process. Complete degradation was evident within 24 h. Attempts to investigate the release kinetics of AGU661 from the NPs were not successful due to the strong hydrophobicity of the drug and consequent precipitation, which hindered reliable quantification of its release profile (Figure S8). Nevertheless, the observed NP degradation will consequently allow the release of AGU661 from the carrier matrix.

**Figure 4:**
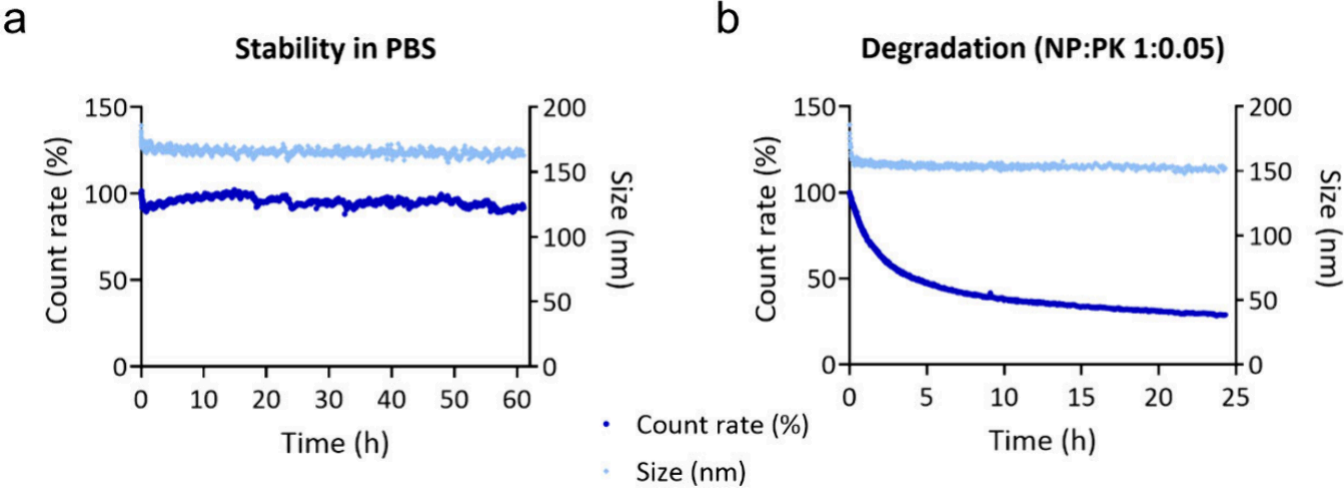
Stability and degradation of PLGA_AGU661._ (a) Stability in PBS and (b) degradation using a NP-to-proteinase K ratio of 1:0.05 monitored at 37 °C by DLS at constant detector settings.

### 3.5 AGU661 NPs Inhibit PGE_2_ Formation in Human Whole Blood

As pointed out above, most mPGES-1 inhibitors fail in human whole blood assays due to their high lipophilicity and, thus, strong unspecific protein binding.^30^ Nevertheless, in blood, the excess of plasma proteins (e.g., albumin) and nontargeted cells like platelets and erythrocytes create a complex environment depicting the (patho-)­physiological situation where the mPGES-1 inhibitor eventually has to operate. Human whole blood that was first stimulated with LPS for 24 h in order to induce COX-2/mPGES-1 expression and thus possesses high capacity to generate PGE_2_ upon activation by SACM (3%). Free AGU661 or AGU661 encapsulated into NPs were added to the LPS-stimulated blood 15 min prior activation with SACM and LM profiles were assessed after 90 min. The LM profiles after modulation by free and encapsulated AGU661 (0.3 to 100 nM) revealed similar patterns, where mainly PGE_2_ was impaired, seemingly more efficient for encapsulated AGU661 (Figure 5a). In more detail, PGE_2_ formation was significantly suppressed starting at 1 nM encapsulated AGU661 with an IC_50_ = 1.5 nM, while significant suppression of PGE_2_ formation by free AGU661 was not obtained up to 100 nM (Figure 5a, b). The blank PLGA NPs (without drug) were used as controls, and these NPs did not affect the overall LM profile of LPS-stimulated blood, excluding immune cell activation or any detrimental effects on blood cells (Figure 5b). Other LMs were not impaired by encapsulated AGU661, except for some moderate suppression of LTB_4_ (Figure 5a). Together, these data clearly indicate that encapsulation of the mPGES-1 inhibitor AGU661 into PLGA-based NPs tremendously improves its potency in whole blood by more than 65-fold versus the free drug.

**Figure 5:**
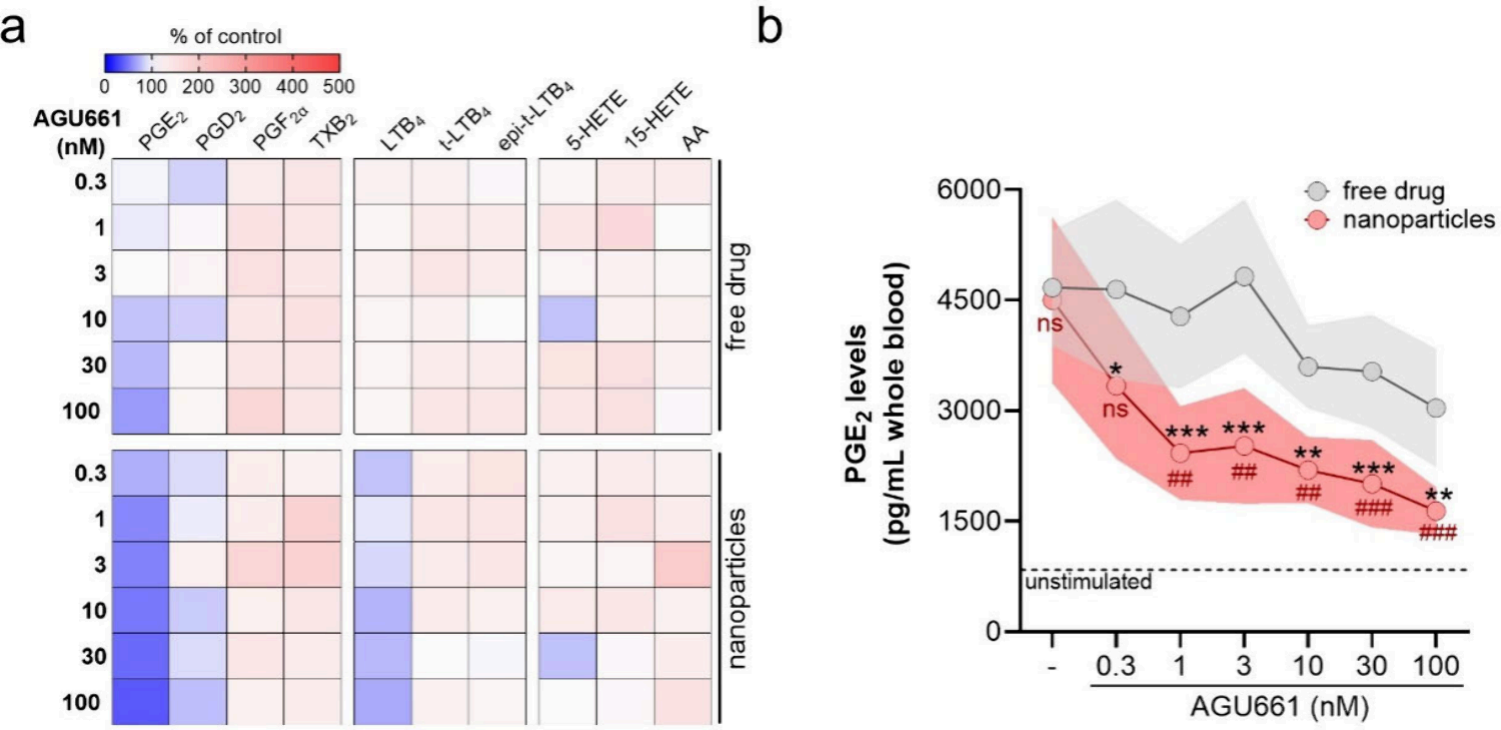
Comparison of free and encapsulated AGU661 to suppress PGE_2_ formation in human whole blood. Human whole blood (0.5 mL) was stimulated with 1 μg/mL LPS for 24 h and then preincubated with vehicle (veh., 0.1% DMSO or blank-loaded NPs), free or encapsulated AGU661 in NPs (0.3, 1, 3, 10, 30, 100 nM) for 15 min and stimulated with 3% SACM for 90 min at 37 °C. Supernatants were collected, and formed LMs were analyzed by UPLC-MS/MS. (a) Results are presented as % of control in a heatmap. (b) PGE_2_ levels are presented as pg/mL human whole blood given as mean ± SEM as colored shadows. *n* = 4. For statistical analysis data were log-transformed and two-way ANOVA with Šídák’s multiple comparison test was performed; * *p* < 0.05, ** *p* < 0.01, *** *p* < 0.001 display comparison between free drug and nanoparticles at same AGU661 concentrations; ns (not significant) *p* > 0.05, # *p* < 0.05, ## *p* < 0.01, ### *p* < 0.001 display comparison between treatment and vehicle.

## 4 Discussion

Current anti-inflammatory strategies, like the use of COX-inhibiting NSAIDs, reduce the formation of all COX-derived PGs, causing several adverse side effects due to suppression of beneficial PGs (e.g., PGD_2_, PGJ_2_ and constitutively formed PGE_2_),^10^ in particular in long-term use.^4,35^ mPGES-1 inhibitors display a more specific class of anti-inflammatory drugs by selectively blocking the massive PGE_2_ production associated with the inducible COX-2/mPGES-1, which results in an improved on-target profile compared to traditional COX inhibitors.^3,5,36^ However, the preclinical development of many mPGES-1 inhibitors failed because of loss of efficiency due to strong plasma protein binding tendency connected to their high lipophilicity.^30^ Here, we present potent cellular activity of the novel mPGES-1 inhibitor AGU661 in pro-inflammatory innate immune cells, and we demonstrate that encapsulation of AGU661 into PLGA-based NPs is suitable to improve its bioavailability upon intravenous application, reflected by the enhanced effectiveness in activated human whole blood. Such a marked gain in efficiency of AGU661 against mPGES-1-mediated PGE_2_ biosynthesis in a pathophysiological environment by drug encapsulation into NPs is a significant advancement in the development of mPGES-1 inhibitors.

We recently identified novel benzimidazole derivatives with the lead compound AGU654 as potent and selective mPGES-1 inhibitors with IC_50_ in the sub- or 1-digit-nanomolar range and high *in vivo* efficacy.^11^ AGU661 was the most potent compound out of this series (IC_50_ = 0.22 nM) with comparable potency to vipoglanstat (IC_50_ = 1 nM), a drug candidate tested in several clinical studies.^37^ In terms of specificity, analysis of AGU661 in several innate immune cells and for interference with isolated enzymes revealed selective inhibition of mPGES-1-mediated PGE_2_ formation, while other PGs (in cells) and COX-1 and COX-2 as isolated enzymes were not inhibited by the drug. The abundance of mPGES-1 and the robust PGE_2_ formation in LPS-primed monocytes and in M1 macrophages elicited by bacterial exotoxins displays a suitable test platform for mPGES-1 inhibitors under pathophysiological relevant conditions.^11^ In fact, AGU661 inhibited cellular PGE_2_ formation only in cells that strongly express mPGES-1, namely in M1-MDM and LPS-treated monocytes, but not in pro-resolving M2-MDM that lack mPGES-1.^29^ The reported shunting effects of mPGES-1 inhibitors toward TXB_2_ and PGI_2_,^38^ were evident for AGU661 only for TXB_2_ with moderate magnitude. In contrast to the dual mPGES-1/FLAP inhibitor BRP-201,^39^ AGU661 did not affect SPM biosynthesis in 15-LOX-expressing M2-MDM, supporting that inhibition of FLAP, but not of mPGES-1, is the driving factor for inducing the LM class switch.^40^ PGE_2_ is known to mediate cytokine release in innate immune cells via its EP1–4 receptors.^41^ Addition of PGE_2_ to macrophages prior LPS stimulation down-regulated pro-inflammatory cytokines,^42^ while addition to untreated macrophages induces minor secretion of IL-6 ^43^ and TNF-α.^44^ However, AGU661 inhibited pro-inflammatory IL-1β, IL-6, and TNF-α in LPS-stimulated monocytes only at high concentrations (100 times higher than the IC_50_ to suppress PGE_2_ levels), implying no direct correlation between the two events.

Experimental inflammation models using mice or rats are commonly used to validate the *in vivo* efficacy of anti-inflammatory drugs.^45,46^ In line with several structural classes of mPGES-1 inhibitors,^5,30^ AGU661 failed to interfere with the murine mPGES-1 ortholog as revealed by its inability to block PGE_2_ formation in LPS/exotoxin-stimulated murine peritoneal macrophages. Therefore, typical mouse inflammation models such as paw edema or peritonitis are unsuitable for evaluating the pharmacological value of AGU661 in mice. To estimate the pharmacological potential of anti-inflammatory drugs, more complex human *ex vivo* models are favorable, such as HWB assays,^47^ because they perfectly depict the pathophysiological environment that a drug has to face upon reaching the systemic circulation. In particular for mPGES-1 inhibitors, there is a distinct correlation between the potency in HWB assays and the effective concentration *in vivo*.^48^ Among the blood cells, monocytes that make up <1% in healthy donors,^49^ exhibit highest mPGES-1 levels after stimulation (e.g., with LPS) and are, thus, the most relevant PGE_2_ producers in HWB, implying them as primary target cells of AGU661. Most mPGES-1 inhibitors compete with the substrate PGH_2_, a lipophilic AA metabolite, and are thus rather lipophilic and prone to unspecific binding to cells and plasma protein as well as to metabolism.^5,30^ AGU661 shares these features and moreover, revealed unfavorable pharmacokinetic features, especially metabolic instability.^11^ Accordingly, the high lipophilicity of AGU661 suggested strong plasma protein binding, which is reflected by the >100-fold loss of potency in HWB versus isolated monocytes in medium/buffer. These detrimental features led us to encapsulate AGU661 into NPs using PLGA as a well-studied and safe polymer by nanoprecipitation.^50^

Encapsulation of small molecule drugs into polymer-based NPs represents a promising strategy that enables overcoming the detrimental physicochemical drug features that impair their bioavailability and efficacy.^51^ For example, encapsulation of lipophilic anti-inflammatory compounds like curcumin or hyperforin,^52^ the dual 5-LOX/mPGES-1 inhibitor BRP-187, or indirubin derivatives into PLGA-based NPs favorably enhances their bioactivity and stability.^53–57^ Exposure of the immune system to NPs can trigger an unwanted activation or suppression of the immune response,^58^ but our findings with blank NPs indicate that they do not trigger immune cell activation. Others have shown that the protein corona, which is formed around the drug-loaded NPs in a biological environment (like HWB), improves the bioavailability of the drug.^54,59,60^ Although we applied such NP encapsulation strategy for the same reasons to the dual mPGES-1/FLAP inhibitor BRP-201 before, solely inhibition of 5-LOX activity but not of PGE_2_ formation could be demonstrated for that compound in blood.^21^

Encapsulation of AGU661 into PLGA-NPs markedly improved the efficiency to suppress PGE_2_ formation in activated HWB. These AGU661-loaded NPs were stable, monodisperse particles with a size of around 180 nm with negative zeta potential, which enables cellular uptake via clathrin-mediated endocytosis.^61^ NPs are actively phagocytized by monocytes in blood^62,63^ which suggest an enrichment of AGU661 in these mPGES-1-expressing target cells. The reasons for the improved efficiency of AGU661 in HWB due to encapsulation might be caused by several processes that act in conjunction: (i) Reduced plasma protein binding of AGU661, (ii) impairment of adsorption and passive uptake of free AGU661 by other (off-target) blood cells, such as erythrocytes, platelets, and lymphocytes that are unable to phagocytose NPs, (iii) a favorable blood protein corona on the NPs connected to better particle uptake, (iv) spatial AGU661 delivery toward mPGES-1 when released from NPs following cellular uptake; in fact, phagocytosis studies revealed that NPs are taken up within 10 min by innate immune cells,^64^ supporting the hypothesis that active uptake and local intracellular supply of AGU661 may govern effective mPGES-1 inhibition. Assessing the uptake of the NP into diverse target cells in blood is not readily doable. Therefore, the assessment of the intracellular bioactivity of AGU661, namely suppression of mPGES-1-mediate PGE_2_ formation within monocytes (the major cells expressing mPGES-1 and producing PGE_2_ in blood under these conditions) is the sole indication for NP internalization in blood.

## 5 Conclusion

Here, we present a prominent approach to enhance the poor bioavailability of lipophilic drugs by their encapsulation into suitable PLGA-based NPs for the potential treatment of inflammatory disorders. We demonstrate that AGU661 is a specific and potent mPGES-1 inhibitor that is highly active in intact innate immune cells but loses effectiveness in HWB, a typical feature of many mPGES-1 inhibitors. To improve the bioavailability and biological activity of AGU661, we encapsulated the drug into solid polymeric NPs based on PLGA using nanoprecipitation, yielding a suitable drug delivery system based on an NP with excellent physicochemical characteristics. Thus, encapsulation of the drug into NPs enhanced the efficiency to suppress PGE_2_ in complex systems, such as exotoxin-stimulated human whole blood. We conclude that encapsulation of AGU661 into PLGA-based NPs significantly enhances its bioactivity under pathophysiologically relevant conditions and encourages more detailed assessment of this pharmacotherapeutic approach for treatment of inflammatory disorders.

## Supplementary Material



## Notes

Formulation parameters for NPs (Table S1) and particle characteristics (Table S2); effects of AGU661 on cell viability in human macrophages (Figure S1) and on LM formation in murine peritoneal macrophages (Figure S2); intensity distributions of purified NPs (Figure S3 and S4) with and without the addition of PVA (Figure S5 and S6); stability and degradation of NPs (Figure S7) and drug release from NPs in proteinase K incubations (Figure S8) plus description of result from stability and drug release studies (PDF)
