# Supplementary material for: Enhanced Efficiency of the Microsomal Prostaglandin E2 Synthase‑1 Inhibitor AGU661 in Human Whole Blood by Encapsulation into PLGA-Based Nanoparticles
Source: Mol Pharm. 2025 Oct 3;22(11):6803–15. doi: 10.1021/acs.molpharmaceut.5c00766 (PMC12587403; doi:10.1021/acs.molpharmaceut.5c00766)
Supplement: Supplementary file 1 [file mp5c00766_si_001.pdf]

Enhanced efficiency of the microsomal prostaglandin E_2 synthase-1 inhibitor AGU661 in human whole blood by encapsulation into PLGA-based nanoparticles

Philipp Dahlke[1,#], Paul M. Jordan[1,2,#], Lea C. Klepsch[2,3], Azize Gizem Ergül[4], Steffi Stumpf[2,3], Stephanie Hoeppener[2,3], Antje Vollrath[2,3], Burcu Çalışkan[4], Erden Banoglu[4], Ulrich S. Schubert[2,3], Oliver Werz[1,2,*]

[1] Department of Pharmaceutical/Medicinal Chemistry, Institute of Pharmacy, Friedrich Schiller University Jena, Philosophenweg 14, 07743 Jena, Germany

[2] Jena Center for Soft Matter (JCSM), Friedrich Schiller University Jena, Philosophenweg 7, 07743 Jena, Germany

[3] Laboratory of Organic and Macromolecular Chemistry (IOMC), Friedrich Schiller University Jena, Humboldtstraße 10, 07743 Jena, Germany

[4] Department of Pharmaceutical Chemistry, Faculty of Pharmacy, Gazi University, Yenimahalle 06560 Ankara, Turkey

[#] Authors contributed equally to the study

* Correspondence: oliver.werz@uni-jena.de; Tel.: +49-3641-9-49801

Supporting Information

Table S1. Formulation parameters for blank- and drug-loaded NPs. S/W, solvent to water ratio.

**Formulation**	**Polymer concentration**	**Drug load**	**Solvent**	**Water phase**	**S/W**
$PLGA_{AGU661}$	15 mg/mL	0.15%	acetone	0.3% PVA	1:8
PLGA	15 mg/mL	-	acetone	0.3% PVA	1:8
$PLGA_{AGU661\ 1\%}$	15 mg/mL	1.5%	acetone	0.3% PVA	1:8

Table S2. Particle characteristics. Loading capacity (LC) in % was assessed by UV-Vis measurements of lyophilized particles. Hydrodynamic diameter (d_h) and polydispersity index (PDI) obtained by DLS in water. Zeta potential was measured post centrifugation in water, PBS, and sodium chloride (NaCl, 0.9v%). All values are shown with n=1.

		After purification				
**Formulation**	**LC [%]**	**d_h [nm]**	**PDI**	**Zeta potential MQ [mV]**	**Zeta potential PBS [mV]**	**Zeta potential NaCl [mV]**
$PLGA_{AGU661}$	0.09	173	0.02	–32.9	–2.8	–0.4
$PLGA_{AGU661}$	0.10	173	0.03	–24.0	–0.8	–3.3
$PLGA_{AGU661\ 1.5\%}$	0.76	173	0.06	–25.6	–2.5	–0.4

Fig. S1.

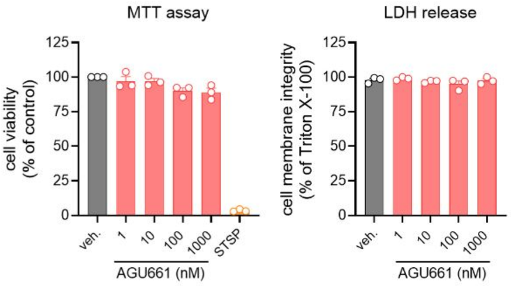

Fig. S1. AGU661 did not alter cell viability in macrophages. Human unpolarized MDM were kept in RPMI 1640 medium containing 10% FCS, L-Glu and penicillin/streptomycin and incubated with vehicle (veh., 0.1% DMSO), or indicated concentrations of AGU661 3 h for LDH assay and 48 h for MTT assay. For MTT assay 1 μM staurosporine (STSP) and for LDH release triton X-100 were used as positive control. Shown values are means + SEM with single values, given as percentage of control, *n* = 3.

Fig. S2.

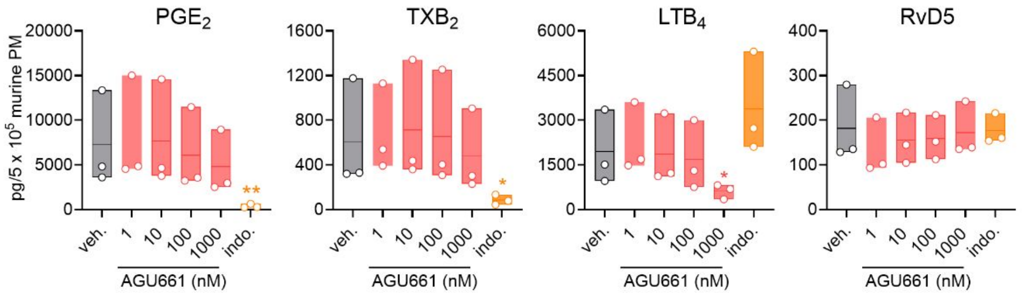

Fig. S2. AGU661 did not affect LM formation in murine peritoneal macrophages. Murine PM (5×10^5) were seeded in RPMI containing 10% FCS, L-Glu and penicillin/streptomycin and prestimulated with 100 ng/mL LPS. Then cells were preincubated with vehicle (veh., 0.1% DMSO), indicated concentrations of AGU661 or indomethacin (indo., 10 µM) for 15 min in 1 mL PBS containing 1 mM $CaCl_2$ and stimulated with 1% SACM for 90 min at 37 °C and 5% CO_2. Results are presented as pg/5×10^5 murine PM as mean with single values, $n = 3$. For statistical analysis data were log-transformed and one-way ANOVA with Dunnett's multiple comparison test against veh. was performed; * $p<0.05$, ** $p<0.01$.

Fig. S3.

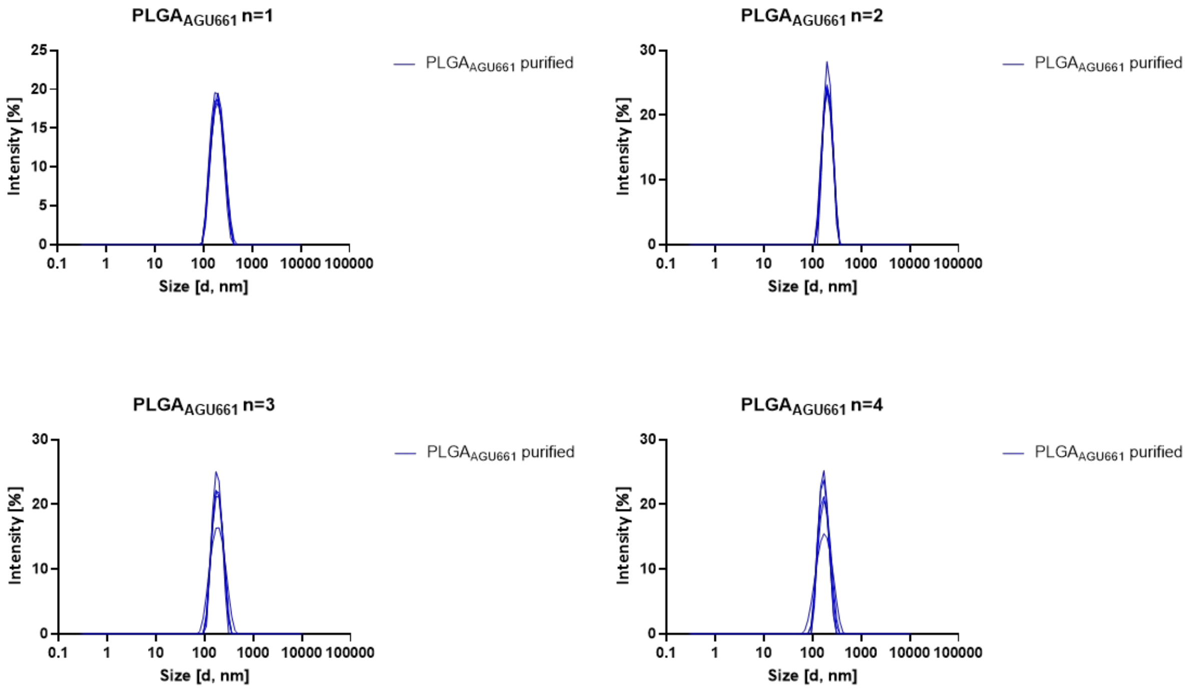

Fig. S3. Intensity distributions of purified PLGA$_{AGU661}$ NPs.

Fig. S4.

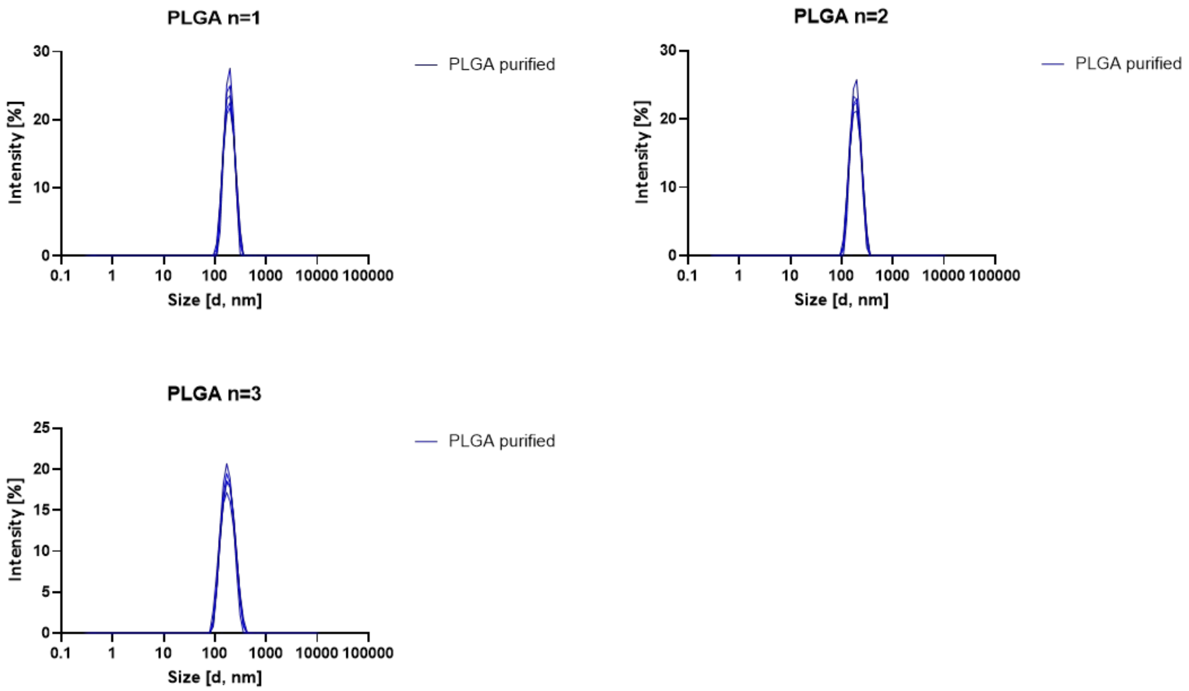

Fig. S4. Intensity distributions of purified PLGA NPs.

Fig. S5.

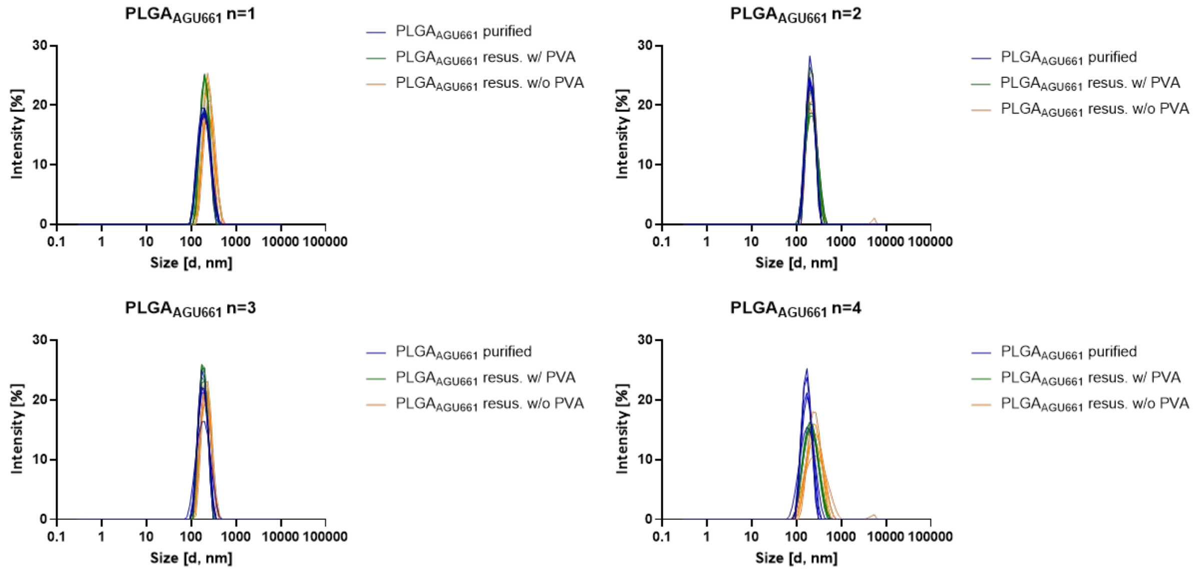

Fig. S5. Intensity distributions of purified PLGA$_{AGU661}$ NPs, resuspended with (w/) and without (w/o) the addition of small amounts of PVA.

Fig. S6.

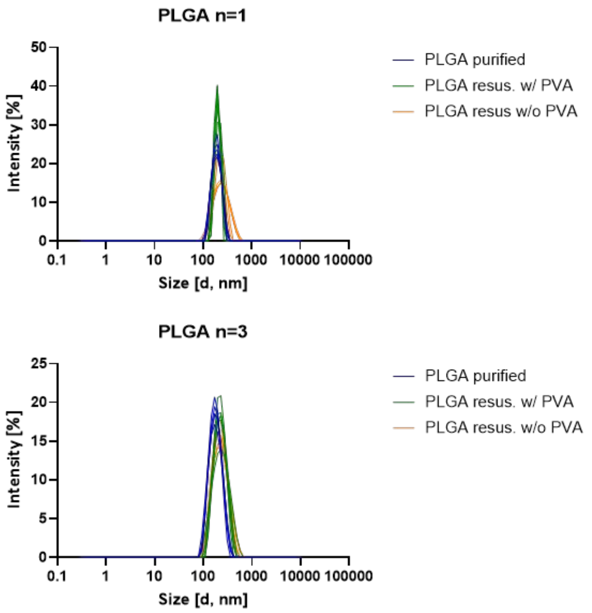

Fig. S6. Intensity distributions of purified PLGA NPs, resuspended with (w/) and without (w/o) the addition of small amounts of PVA.

Fig. S7.

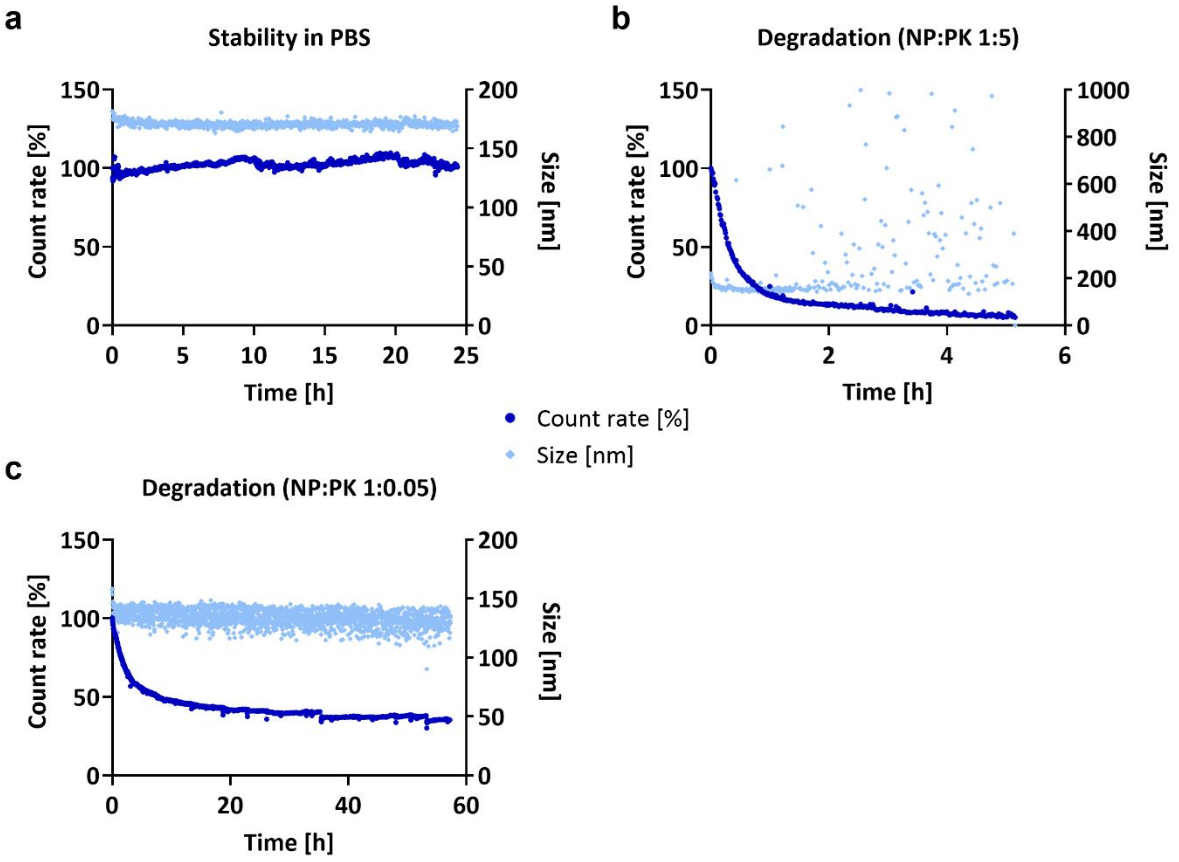

Fig. S7. Stability and degradation of 1% loaded PLGA$_{AGU661}$ NPs (**a**) Stability in PBS and (**b-c**) degradation using two different nanoparticle-to-proteinase K ratios monitored at 37 °C by DLS at constant detector settings.

Fig. S8.

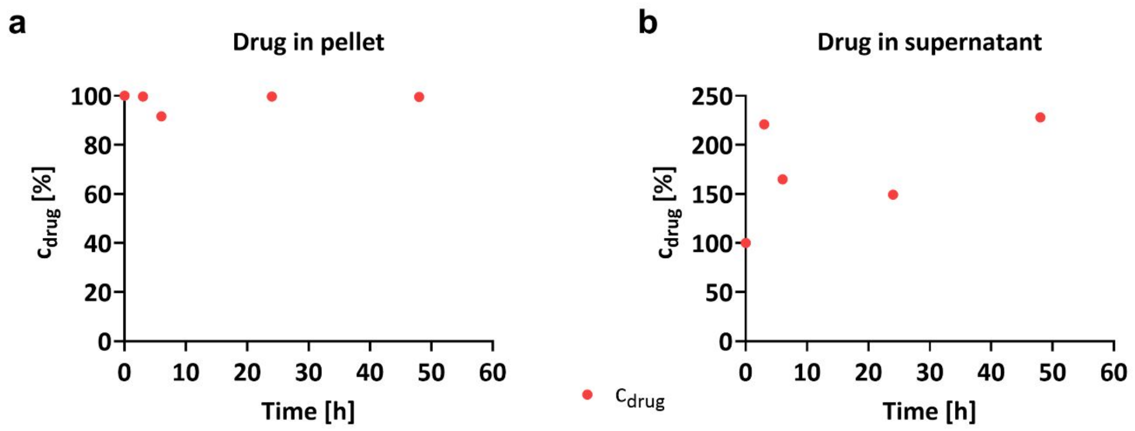

Fig. S8. Drug release of 1% loaded $PLGA_{AGU661}$ NPs in proteinase K (NP:PK 1:0.05) incubations. (**a**) Drug concentration detected in pellet in percent and (**b**) in the supernatant in percent.

Supporting Results

Stability studies of $PLGA_{AGU661\ 1.5\%}$ NPs

To enable drug release studies, NPs with a higher drug loading of 1.5% AGU661 were formulated, allowing more pronounced changes in the drug load over time. As an initial step, the stability of these higher drug-loaded NPs was assessed to confirm similar results obtained with the previously characterized $PLGA_{AGU661}$ particles.

For this purpose, $PLGA_{AGU661\ 1.5\%}$ NPs were incubated in PBS at 37 °C and analyzed by DLS under constant detector settings to track changes in count rate and hydrodynamic diameter. As shown in **Fig. S7a**, both parameters remained stable for up to 24 h, confirming colloidal stability under these conditions. To evaluate enzymatic degradation, proteinase K was selected as a model enzyme, enabling comparison with $PLGA_{AGU661}$ NPs in this study. $PLGA_{AGU661\ 1.5\%}$ NPs were therefore incubated with proteinase K solutions at two nanoparticle-to-enzyme mass ratios (1:5 and 1:0.05; **Fig. S7b,c**). In both cases, a pronounced decrease in count rate was observed, indicating particle degradation, while no increase in hydrodynamic diameter was detected, excluding particle aggregation. The decay of the count rate and as such the apparent particle degradation was thereby dependent on the overall enzyme concentration applied while higher enzyme concentration lead to a faster decrease in the particle count rate.

Drug release studies

For drug release studies, the nanoparticle-to-enzyme ratio of 1:0.05 was selected, as this condition exhibited the slowest degradation. Five aliquots of the same NP dispersion in proteinase K incubations were treated at 37 °C and collected at five different time points. At each time point, dispersions were centrifuged (20 °C, 14,000 rpm, 30 min), and both the supernatant and the resuspended pellet were subsequently freeze-dried. The AGU661 content of all lyophilized samples was quantified using the same UV-Vis method employed for initial drug loading determination, with the drug concentration at time zero (0 h) normalized to 100%. As shown in **Fig. S8**, the drug content in the pellet remained nearly constant throughout the study, whereas the amount detected in the supernatant increased over time, albeit with substantial fluctuations. Considering the rapid decay of the particle count rate observed in the degradation experiments, efficient drug release within several hours was expected. However, the data highlight the challenge in assessing the release of highly hydrophobic drugs. Upon exposure to aqueous environments, such drugs tend to precipitate once no longer stabilized by a hydrophobic matrix or surfactant. Consequently, free hydrophobic drug molecules cannot be readily separated into the aqueous phase by conventional methods such as dialysis or centrifugation. Instead, they are prone to associate with hydrophobic polymer domains or dialysis membranes, or to aggregate and co-precipitate with the NPs, which makes reliable quantification of drug release challenging.
